# Therapeutic Augmentation of Intra-articular Hyaluronic Acid With Triamcinolone Acetonide and Lignocaine in the Management of Primary Knee Osteoarthritis: A Prospective Comparative Study

**DOI:** 10.7759/cureus.115124

**Published:** 2026-08-24

**Authors:** Ananta N Panda, Sanket Mishra, Shubhranshu Choudhary, Deepak Nemani, Subrat Mohapatra, Adish Mittal

**Affiliations:** 1 Department of Orthopaedics, Institute of Medical Sciences and SUM Hospital, Bhubaneswar, IND

**Keywords:** analgesic consumption, hyaluronic acid, knee osteoarthritis, lignocaine, range of motion, triamcinolone acetonide, viscosupplementation, visual analogue scale

## Abstract

Introduction: Primary osteoarthritis (OA) of the knee is a very common cause of pain and disability among the general population with advancing age. Intra-articular hyaluronic acid (HA) injections are widely used for symptomatic relief, while corticosteroids are known to provide rapid anti-inflammatory effects. This study compared intra-articular HA alone with HA combined with triamcinolone acetonide and lignocaine, with the primary objective of comparing the between-group change in Visual Analogue Scale (VAS) pain score from baseline to 12 months and providing a viable conservative treatment option for patients not immediately requiring surgical intervention.

Methods: A prospective comparative study was conducted over 18 months. A* *total of 354 patients with symptomatic primary knee OA were enrolled in this nonrandomized prospective comparative study and allocated to two treatment groups using an alternate (quasi-randomized) allocation sequence based on sequential order of enrollment. After exclusions, 320 patients completed the study (Group A: HA alone, n=162; Group B: HA + triamcinolone acetonide + lignocaine, n=158). Patients were followed at three weeks, six weeks, three months, six months, and 12 months. Pain was assessed using the VAS, knee range of motion measurement, and analgesic consumption profile, while patient satisfaction and adverse events were also recorded.

Results: The mean VAS fell from 7.5±0.9 to 5.4±0.8 in Group A and from 7.6±0.8 to 3.4±0.7 in Group B by 12 months (between-group difference in change 2.1, p<0.001). At 12 months, fewer Group B patients required daily analgesics (29.1% vs 48.8%, p<0.001), and more reported excellent or good satisfaction (83.0% vs 60.5%, p<0.001), while adverse events were infrequent and comparable between groups aside from post-injection knee pain, which was less frequent in Group B (8.2% vs 17.3%, p=0.015).

Conclusion: The addition of triamcinolone acetonide and lignocaine to intra-articular HA was associated with earlier pain relief, improved knee range of motion, reduced analgesic dependence, and higher patient satisfaction without increasing treatment-related complications. Combination therapy appears to be a safe and effective conservative treatment option for symptomatic primary knee OA not immediately requiring surgical intervention. However, because each arm was administered by a single, different surgeon, treatment and operator effects are fully confounded, and findings should be interpreted as reflecting the combined protocol rather than the drug regimen in isolation.

## Introduction

Osteoarthritis (OA) of the knee is one of the most common chronic musculoskeletal disorders worldwide and is a leading cause of pain, disability, and reduced quality of life, particularly among the elderly population [1]. The disease is characterized by progressive attrition of articular cartilage, subchondral bone remodeling, synovial inflammation, and osteophyte formation, resulting in progressive pain, stiffness, and limitation of daily activities [2]. With increasing life expectancy, obesity and sedentary lifestyles, the prevalence of knee OA continues to rise, creating a substantial socioeconomic burden [3].

Management of knee OA is multimodal and includes patient education, weight reduction, physiotherapy, oral analgesics, non-steroidal anti-inflammatory drugs, intra-articular injections and, in advanced cases, total knee arthroplasty. As many patients either are not suitable surgical candidates or wish to postpone surgery, effective conservative treatment options remain an important component of disease management.

Intra-articular hyaluronic acid (HA) has been widely used as a viscosupplement because it restores the viscoelastic properties of synovial fluid, improves joint lubrication, and may provide prolonged symptomatic relief. However, its onset of action is relatively slow, and the magnitude of clinical benefit varies among patients. In contrast, intra-articular corticosteroids provide rapid reduction of synovial inflammation and pain, but their effect is generally short-lived.

Combining HA with a corticosteroid and a local anaesthetic may offer the advantages of both therapies. Triamcinolone acetonide can provide rapid suppression of inflammation, while lignocaine offers immediate procedural analgesia and improved patient comfort. HA may subsequently maintain symptom relief through its viscosupplementation effect. Several randomized trials and meta-analyses have compared HA with corticosteroids as competing treatments, and some have examined combined corticosteroid-HA regimens; fewer have specifically evaluated a single-injection combination incorporating a local anesthetic alongside these two agents.

Although numerous nonsurgical treatment options for knee OA have been investigated, including platelet-rich plasma, prolotherapy, microfragmented adipose tissue, extracorporeal shock wave therapy, hydrodissection and other image-guided or regenerative interventions, these approaches often require specialized equipment, additional operator training, or greater cost, limiting their accessibility in routine clinical practice, particularly in resource-limited settings. HA and corticosteroids, in contrast, are already widely available and routinely used in everyday orthopedic practice. The knowledge gap addressed by this study is therefore practical rather than exploratory: whether combining these two already-accessible agents with a local anesthetic in a single injection can improve outcomes achievable with either agent alone, using resources already available in routine clinical settings. The present prospective comparative study was therefore undertaken to compare a single intra-articular injection of HA alone with HA combined with triamcinolone acetonide and lignocaine in patients with symptomatic primary OA of the knee. The primary objective was to compare the between-group change in Visual Analogue Scale (VAS) pain score from baseline to 12 months. Secondary objectives included between-group differences in knee range of motion (ROM), analgesic consumption, patient satisfaction, and treatment-related adverse events over the same follow-up period.

## Materials and methods

Study design

This prospective comparative study was conducted in the Department of Orthopaedics, Institute of Medical Sciences and SUM Hospital, Bhubaneswar, Odisha, India. Patient enrollment occurred over approximately five months, from 1 October 2024 to February 2025, after which no further patients were enrolled. All enrolled patients were followed for 12 months from the time of injection, with final follow-up visits completed by 31 March 2026, giving a total study period of 18 months. The study was designed to compare the short- to mid-term functional outcomes following a single intra-articular injection of HA alone versus HA combined with triamcinolone and lignocaine in patients with symptomatic primary OA of the knee. Sample size was determined by the fixed study duration and follow-up requirement rather than by a priori power calculation. Enrollment continued until the last date by which a newly enrolled patient could still complete 12-month follow-up within the 18-month study period, at which point enrollment was closed. This was a nonrandomized prospective comparative cohort study using alternate (quasi-randomized) allocation rather than formal randomization. The study was not prospectively registered in a clinical trials registry.

Patient selection

Patients attending the orthopedic outpatient department with symptomatic primary knee OA who fulfilled the eligibility criteria were invited to participate. Written informed consent was obtained before enrollment. For patients with bilateral knee involvement, the knee with more severe or dominant symptoms, as identified by the patient, was selected for treatment and study inclusion; the knee was therefore the unit of analysis, with one knee analyzed per patient.

A total of 354 patients were enrolled and allocated to one of two treatment groups using a pre-defined alternate allocation sequence (described below). The number of patients screened for eligibility prior to enrollment, and reasons for any pre-enrollment exclusions, were not separately recorded. During follow-up, 16 patients were lost to follow-up, 11 patients failed to comply with the study protocol, and seven patients underwent additional traditional or alternative treatments during the study period and were excluded. Consequently, 320 patients completed the study and were included in the final analysis. Group A consisted of 162 patients, while Group B consisted of 158 patients.

Inclusion criteria

The inclusion criteria included (1) age >40 years; (2) symptomatic primary OA of the knee; (3) Kellgren-Lawrence Grade II, III, or IV; (4) persistent symptoms despite conservative medical treatment; (5) ability to comply with follow-up; and (6) written informed consent.

Exclusion criteria

The following criteria were excluded from our study: (1) inflammatory arthritis, (2) septic arthritis, (3) previous surgery on the affected knee, (4) advanced OA requiring urgent arthroplasty, (5) recent intra-articular injection within the previous three months, (6) coagulopathy or active infection, (7) failure to complete follow-up, (8) use of additional injection therapy or traditional/alternative treatment during the study period, and (9) bilateral knee involvement with inability to identify a single dominantly symptomatic knee.

Group allocation

Patients were assessed for eligibility and consented for both injection therapy and study participation by the study team at the orthopedic OPD. Allocation to treatment group followed a systematic alternation sequence based on the sequential order of enrollment: patients were assigned alternately to Group A and Group B in the order in which they were enrolled and consented (first, third, fifth... enrolled patient to Group A; second, fourth, sixth... to Group B). This represents a quasi-randomization (alternate allocation) method rather than formal randomization, as the sequence was predictable and allocation was not concealed from the study team at the point of assessment. Following assignment, Group A patients were referred to a consultant orthopedic surgeon who administered intra-articular HA alone, and Group B patients to a second consultant orthopedic surgeon who administered combined intra-articular HA, triamcinolone, and lignocaine. Both consultants served solely as treating physicians for injection administration and were not informed of the study; they had no role in patient selection, consent, or group assignment, which were determined entirely by the study team prior to referral.

Injection protocol

Group A

Group A received Halonix® (Cadila Pharmaceuticals, manufactured by Heil Pharma, Ahmedabad, India), a sodium HA injection (1.5% w/v) supplied as sterile 2 mL prefilled syringes for intra-articular use only. Each mL contained 15 mg of sodium hyaluronate EP in phosphate-buffered saline. Group A received a total volume of 4 mL (two prefilled syringes; 60 mg of sodium hyaluronate), administered successively using the same 21-gauge needle provided in the pack without changing the needle position or replacing the needle.

Group B

Patients received the same intra-articular injection consisting of Halonix® 4 mL (two prefilled syringes; 60 mg sodium hyaluronate), triamcinolone acetonide (Kenacort®; Abbott Laboratories, Chicago, Illinois, USA) 40 mg in 1 mL, and 2% lignocaine (Lox®; Neon Laboratories Ltd., Mumbai, Maharashtra, India) 1 mL, with a total injected volume of 6 mL. For Group B, triamcinolone acetonide and lignocaine were combined in a single syringe and injected first, followed by HA from a separate prefilled syringe; both were administered sequentially through the same 21-gauge needle placement without repositioning.

All injections were administered under strict aseptic precautions using the standard anterolateral approach to the knee joint. A landmark-guided anterolateral approach was used rather than ultrasound guidance, as this technique is widely accessible, does not require specialized equipment or additional operator training, and is standard practice for intra-articular knee injection at our institution. Where a significant joint effusion was present, aspiration was performed prior to injection as standard departmental protocol in both groups. Both surgeons used the same anterolateral approach, sterilization setup, and equipment as described above; however, no formal checklist or inter-operator standardization assessment was used to verify procedural consistency between surgeons.

Follow-up

Patients were evaluated at baseline, three weeks, six weeks, three months, six months, and 12 months. At each visit, the VAS, knee ROM, analgesic requirement, patient satisfaction, and procedure-related complications were recorded. The primary outcome measure was improvement in VAS (pain) score. Secondary outcome measures included changes in knee ROM, analgesic consumption, patient satisfaction, and adverse events. No formal restriction was placed on physiotherapy during the study period. Home-based exercise was advised and encouraged equally in both groups. Consistent with the exclusion criteria, patients were advised against additional traditional or allied medical treatment, further intra-articular injections, or other adjunct therapies such as chiropractic or acupuncture during the study period.

Outcome measurement

Radiographic severity of OA was graded using the Kellgren-Lawrence classification system [4]. Pain intensity was assessed using the VAS, a 10-cm horizontal line on which patients marked their level of pain from 0 (no pain) to 10 (worst imaginable pain) [5]. Knee ROM was measured in degrees of flexion using a standard goniometer. Analgesic consumption was recorded as daily requirement of oral analgesics (yes/no). Patient satisfaction was recorded using a non-validated four-point ordinal scale (excellent, good, fair, poor). For patients not literate in English, the scale was administered using standardized Odia equivalents obtained via machine translation (Excellent - Utkrusta/Bahut Bhala; Good - Bhala Laguchi; Fair - Thik Thak; Poor - Kharap). Most patients understood and responded directly using these terms; for the minority who did not, a brief additional verbal explanation was provided by the assessor. Outcome assessors responsible for VAS scoring, goniometric ROM measurement, and Kellgren-Lawrence grading were aware that a study was in progress but had no access to the enrollment or allocation database and were blinded to individual patients' treatment group assignment. Treating surgeons administered injections as part of routine outpatient care and were not informed of the study or its objectives.

Statistical analysis

Data were entered into Microsoft Excel (Microsoft Corp., Redmond, WA, USA) and analysed using IBM SPSS Statistics for Windows, Version 31 (Released 2025; IBM Corp., Armonk, New York, United States). Continuous variables were expressed as mean ± standard deviation (SD) and categorical variables as frequencies and percentages. Between-group comparisons at each follow-up time point were performed using the independent Student's t-test for continuous variables and the chi-square test for categorical variables. A p-value <0.05 was considered statistically significant. Effect sizes were not formally calculated for continuous outcomes; between-group mean differences with their associated t-statistics are reported to allow readers to gauge the magnitude of effect alongside statistical significance. All statistical tests were two-tailed. Missing data arising from loss to follow-up were not imputed; analyses were restricted to patients with complete follow-up data (per-protocol population), as detailed in the Limitations section.

## Results

**Figure 1:**
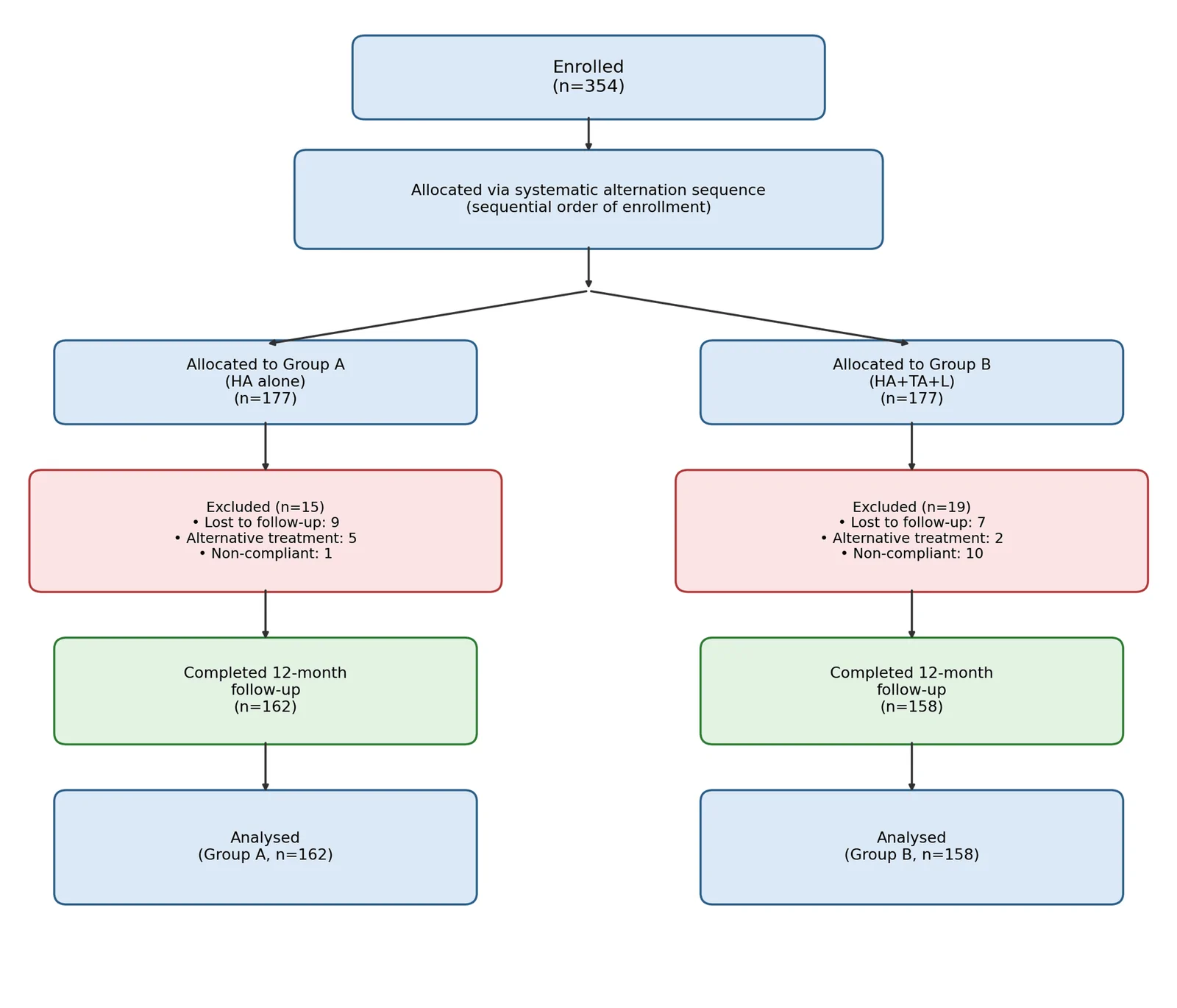
Participant flow diagram HA: hyaluronic acid; TA: triamcinolone acetonide; L: lignocaine

Of the 354 patients enrolled (177 allocated to each group via the alternation sequence), 34 did not complete the study. In Group A, 15 patients were excluded (nine lost to follow-up, five who received additional alternative treatment, and one non-compliant with the protocol). In Group B, 19 patients were excluded (seven lost to follow-up, two who received additional alternative treatment, and 10 non-compliant with the protocol). The remaining 320 patients completed the 12-month follow-up: 162 in Group A and 158 in Group B (Table 1). The patient flow diagram is shown in Figure 1.

**Table 1:** Arm-specific attrition during follow-up

Reason for exclusion	Group A (n=177)	Group B (n=177)	Total
Lost to follow-up	9	7	16
Additional alternative treatment	5	2	7
Non-compliant with protocol	1	10	11
Total excluded	15	19	34
Completed study	162	158	320

Baseline demographic and clinical characteristics

The two groups were comparable at baseline with respect to age, sex distribution, side of knee involvement, and duration of symptoms, with no statistically significant differences. BMI was marginally higher in Group A than in Group B (25.2±1.6 vs 24.8±1.8 kg/m^2^, t(318)=2.10, p=0.04); although this difference reached statistical significance, the absolute difference of only 0.4 kg/m^2^ is well below any threshold regarded as clinically meaningful and both group means remained within the same BMI category, making a confounding effect on treatment outcomes unlikely (Table 2).

**Table 2:** Baseline demographic characteristics An independent Student's t-test was used for continuous variables (age, BMI, duration of symptoms); a chi-square test was used for categorical variables (sex, laterality).

Variable	Group A (n=162)	Group B (n=158)	Test statistic	p-value
Mean age (years)	58.4±7.2	57.6±6.9	t(318)=1.01	0.32
Female	106 (65.4%)	111 (70.3%)	χ^2^(1)=0.85	0.35
Male	56 (34.6%)	47 (29.7%)	-	-
BMI (kg/m^2^)	25.2±1.6	24.8±1.8	t(318)=2.10	0.04
Right knee	88 (54.3%)	85 (53.8%)	χ^2^(1)=0.01	0.93
Left knee	74 (45.7%)	73 (46.2%)	-	-
Duration of symptoms (months)	24.4±10.3	25.2±9.8	t(318)=0.71	0.47

Radiological grading of OA severity (Kellgren-Lawrence grade) was also similar between the two groups, with no significant difference in the distribution of Grade II, III, or IV disease (Table 3).

**Table 3:** Radiological (Kellgren-Lawrence) grading A chi-square test was used to compare the overall distribution of Kellgren-Lawrence grades between groups.

Grade	Group A	Group B	Test statistic	p-value
Grade II	65 (40.1%)	60 (38.0%)	-	-
Grade III	87 (53.7%)	89 (56.3%)	-	-
Grade IV	10 (6.2%)	9 (5.7%)	χ^2^(2)=0.23	0.89

Baseline clinical parameters, including VAS score, knee ROM, daily analgesic use, and presence of knee effusion, were similar between groups prior to treatment, confirming comparability of the two cohorts at study entry (Table 4).

**Table 4:** Baseline clinical parameters An independent Student's t-test was used for continuous variables (VAS, knee flexion); a chi-square test was used for categorical variables (daily analgesic use, knee effusion). VAS: Visual Analogue Scale; NS: not significant

Variable	Group A	Group B	Test statistic	p-value
VAS	7.5±0.9	7.6±0.8	t(318)=1.05	0.321
Knee flexion	111.8±8.6°	112.2±8.4°	t(318)=0.42	0.674
Daily analgesic use	156 (96.3%)	152 (96.2%)	χ^2^(1)=0.00	NS
Knee effusion	98 (60.5%)	94 (59.5%)	χ^2^(1)=0.03	0.864

Pain relief (VAS score)

**Figure 2:**
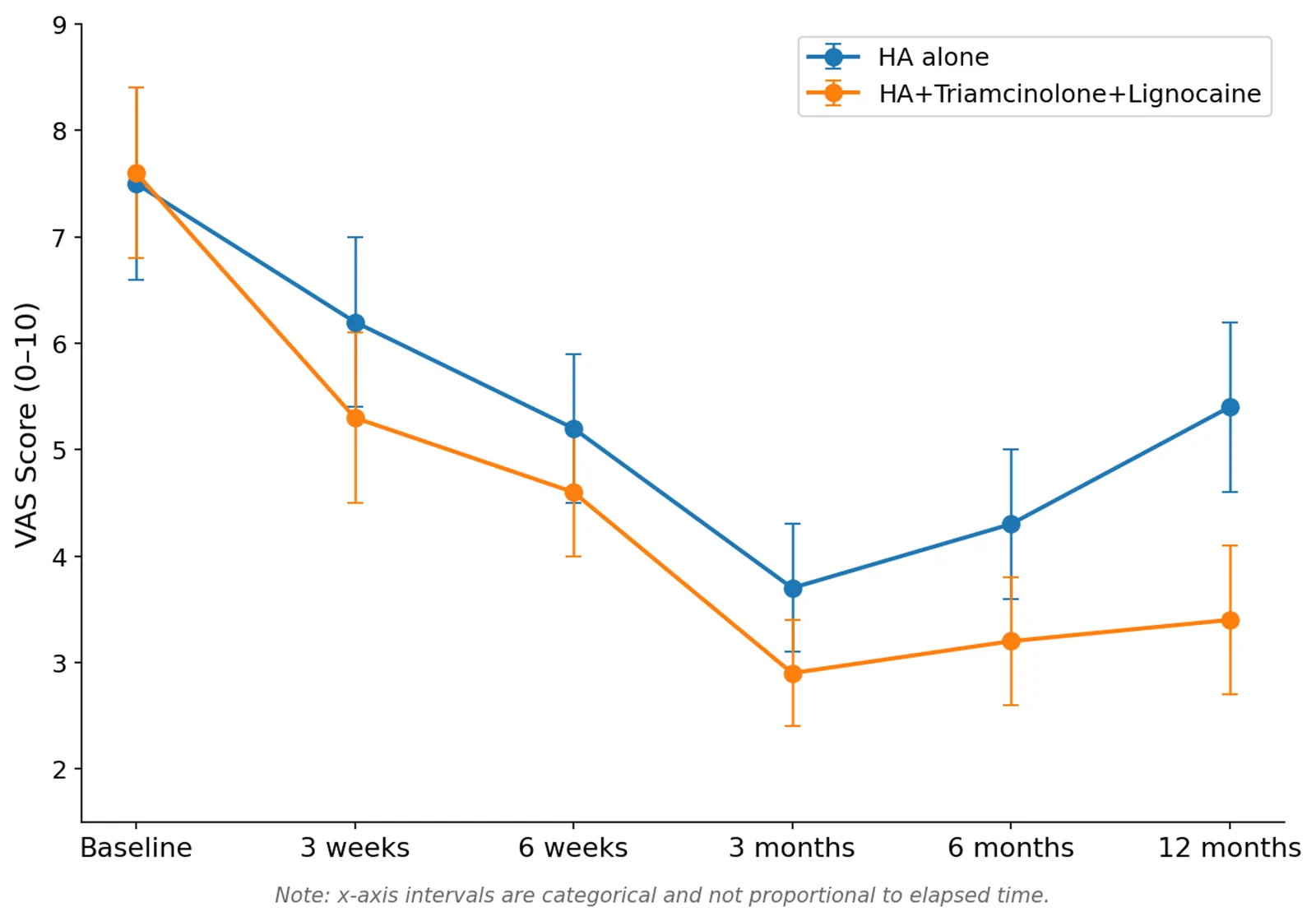
Trend of VAS score over the 12-month follow-up period in Group A and Group B VAS: Visual Analogue Scale; HA: hyaluronic acid

Both groups showed a progressive reduction in VAS score following injection, with the greatest improvement observed at three months in both groups, followed by a gradual increase up to 12 months. From the three-week assessment onwards, Group B demonstrated significantly lower VAS scores than Group A at every follow-up time point (p<0.001), and this advantage was maintained through 12 months (Tables 5-6 and Figure 2).

**Table 5:** Visual Analogue Scale (VAS) score at each follow-up visit An independent Student's t-test was used at each follow-up time point.

Follow-up	Group A	Group B	Test statistic	p-value
Baseline	7.5±0.9	7.6±0.8	t(318)=1.05	0.321
Three weeks	6.2±0.8	5.3±0.8	t(318)=10.06	<0.001
Six weeks	5.2±0.7	4.6±0.6	t(318)=8.22	<0.001
Three months	3.7±0.6	2.9±0.5	t(318)=12.94	<0.001
Six months	4.3±0.7	3.2±0.6	t(318)=15.08	<0.001
12 months	5.4±0.8	3.4±0.7	t(318)=23.78	<0.001

**Table 6:** Change in VAS score from baseline to 12 months * Confidence intervals and p-values for the change scores are approximate, derived by propagating the baseline and 12-month group-level standard deviations (SDs) as if independent (SD_change = √(SD_baseline^2^ + SD_12mo^2^)). Because baseline and 12-month scores in the same patient are expected to be positively correlated, this method is conservative and modestly overestimates the true variance of the change score; the true 95% CI is expected to be narrower than shown. VAS: Visual Analogue Scale; CI: confidence interval

Group	Baseline VAS	12-month VAS	Mean change (95% CI)*	p-value*
Group A (n=162)	7.5±0.9	5.4±0.8	2.1 (1.91 to 2.29)	<0.001
Group B (n=158)	7.6±0.8	3.4±0.7	4.2 (4.03 to 4.37)	<0.001
Between-group difference in change	-	-	2.1 (1.85 to 2.35)	<0.001

Knee ROM

Both groups demonstrated progressive improvement in knee flexion following injection. From the three-week assessment onwards, Group B showed significantly greater improvement in knee ROM compared to Group A at every follow-up time point (independent Student's t-test, p<0.001 from three weeks onwards), with superior ROM maintained through 12 months (Table 7).

**Table 7:** Knee range of motion (ROM) during follow-up An independent Student's t-test was used at each follow-up time point. NS: not significant

Follow-up	Group A (n=162)	Group B (n=158)	Test statistic	p-value
Baseline	111.8±8.6°	112.2±8.4°	t(318)=0.42	NS
Three weeks	114.3±5.9°	117.8±5.5°	t(318)=5.49	<0.001
Six weeks	117.4±5.3°	120.9±4.8°	t(318)=6.19	<0.001
Three months	120.8±6.7°	124.8±6.2°	t(318)=5.54	<0.001
Six months	119.3±5.9°	123.2±6.5°	t(318)=5.62	<0.001
12 months	117.1±6.1°	120.4±5.8°	t(318)=4.96	<0.001

Analgesic consumption

Nearly all patients (96%) required daily analgesics at baseline due to chronic knee pain. Both groups showed significant reductions in analgesic consumption following treatment. From the three-week assessment onwards, Group B demonstrated significantly lower rates of daily analgesic use compared to Group A at every follow-up time point (chi-square test, p<0.001 at all time points), with this advantage maintained through 12 months. By 12 months, 48.8% (n=79) of Group A patients remained on daily analgesics compared to only 29.1% (n=46) in Group B, representing a 69.7% reduction in medication dependence for the combination therapy group versus 49% for monotherapy (Table 8).

**Table 8:** Analgesic consumption during follow-up A chi-square test was used at each follow-up time point to compare proportions of patients requiring daily analgesic use between groups. NS: not significant

Follow-up	Group A (n=162)	Group B (n=158)	Test statistic	p-value
Baseline	156 (96.3%)	152 (96.2%)	χ^2^(1)=0.00	NS
Three weeks	104 (64.2%)	71 (44.9%)	χ^2^(1)=11.97	<0.001
Six weeks	81 (50.0%)	47 (29.7%)	χ^2^(1)=13.67	<0.001
Three months	56 (34.6%)	26 (16.5%)	χ^2^(1)=13.77	<0.001
Six months	68 (42.0%)	34 (21.5%)	χ^2^(1)=15.41	<0.001
12 months	79 (48.8%)	46 (29.1%)	χ^2^(1)=12.98	<0.001

Patient satisfaction

At final follow-up, a significantly greater proportion of patients in Group B reported excellent (n=62 cases) or good (n=69 cases) outcomes (83.0% combined) compared with Group A (60.5% combined) (n=34 excellent and n=64 good), while poor outcomes were less frequent in Group B 3.8% (n=6) versus 11.1% (n=18) (p<0.001) (Table 9).

**Table 9:** Patient-reported outcome at final follow-up A chi-square test was used to compare the overall distribution of outcome categories (excellent/good/fair/poor) between groups.

Outcome	Group A	Group B	Test statistic	p-value
Excellent	34 (21.0%)	62 (39.2%)	-	-
Good	64 (39.5%)	69 (43.7%)	-	-
Fair	46 (28.4%)	21 (13.3%)	-	-
Poor	18 (11.1%)	6 (3.8%)	χ^2^(3)=23.64	<0.001

Adverse events

Both treatment protocols were well tolerated. Post-injection knee pain was significantly less frequent in Group B (13 cases) than in Group A (28 cases) (8.2% versus 17.3%, p=0.015). Transient knee swelling, local tenderness, and injection-site erythema occurred at similar rates in both groups, with no statistically significant differences. No cases of septic arthritis or allergic reaction were recorded in either group (Table 10).

**Table 10:** Procedure-related adverse events A chi-square test was used for each adverse event category, except injection-site erythema, for which Fisher's exact test was used due to expected cell counts below 5.

Adverse event	Group A	Group B	Test statistic	p-value
Post-injection knee pain	28 (17.3%)	13 (8.2%)	χ^2^(1)=5.87	0.015
Transient knee swelling	15 (9.3%)	8 (5.1%)	χ^2^(1)=2.11	0.146
Local tenderness	22 (13.6%)	18 (11.4%)	χ^2^(1)=0.35	0.553
Injection-site erythema	3 (1.9%)	2 (1.3%)	Fisher's exact	1.00
Septic arthritis	0	0	-	-
Allergic reaction	0	0	-	-

## Discussion

The present prospective comparative study demonstrated that the addition of triamcinolone and lignocaine to intra-articular HA was associated with superior pain relief, greater functional improvement, and higher patient satisfaction compared with HA alone. Baseline demographic characteristics, radiological severity, and clinical parameters were comparable between the two groups, aside from a statistically significant but clinically negligible 0.4 kg/m^2^ difference in BMI, suggesting that baseline imbalance alone is unlikely to fully account for the observed between-group differences, although the nonrandomized design cannot exclude the influence of unmeasured confounding factors.

Pain reduction was evident in both groups following treatment; however, patients receiving combination therapy experienced significantly greater improvement from the third week onwards, with maximum benefit observed at three months and sustained superiority up to 12 months. Bannuru et al. demonstrated that corticosteroids provide greater pain relief during the first four weeks, whereas HA becomes more effective beyond eight weeks and maintains benefit over a longer duration [6]. Wang and He reached similar conclusions in their meta-analysis, reporting superior early efficacy with corticosteroids and more durable benefit with HA [7]. Unlike these comparative studies, our protocol combined both agents in a single injection. The sustained reduction in VAS scores observed in our patients is consistent with the hypothesis that the rapid anti-inflammatory effect of triamcinolone may complement the longer-lasting viscoelastic and chondroprotective properties of HA; however, this mechanism was not directly assessed in this study and remains speculative.

Regarding the lidocaine component, the final estimated concentration in the injectate was approximately 0.33% (1 mL of 2% lidocaine in a 6 mL total injected volume), which is further diluted upon mixing with any pre-existing synovial fluid. This concentration is likely below that required for a reliable, sustained sensory nerve block. However, the inclusion of a local anesthetic when mixing with a corticosteroid suspension is standard clinical practice for reasons independent of achieving full anesthetic block: it provides brief procedural comfort, confirms accurate intra-articular needle placement through immediate patient sensation, and improves dilution and diffusion of the crystalline triamcinolone suspension through the joint space [8]. Higher lidocaine concentrations were deliberately avoided given the concentration-dependent chondrotoxic potential of local anesthetics reported in the literature. We therefore do not attribute the observed between-group differences in VAS, satisfaction, or functional outcomes to a direct anesthetic nerve-blocking effect of lidocaine at this concentration; any procedural-comfort or diffusion-related contribution cannot be quantified from this study's design.

Notably, the between-group VAS gap widened rather than narrowed over the follow-up period, which is not fully explained by the short-acting corticosteroid mechanism proposed above; operator effects, the unblinded design, and the differential attrition described in the Limitations may have contributed.

The superior pain relief observed in the combination therapy group was accompanied by significantly greater improvement in knee ROM and a progressive reduction in analgesic consumption throughout the follow-up period. Patients receiving combined drug therapy showed improved restoration of knee mobility with sustained improvement during follow-up, suggesting that rapid suppression of synovial inflammation facilitated improved joint function. Although previous studies have primarily focused on pain and patient-reported functional scores, improvement in knee ROM has also been reported following successful intra-articular HA therapy and is generally considered an indirect indicator of reduced pain and inflammation [9]. Likewise, the significantly lower requirement for rescue analgesics observed in the combination therapy group reflects superior symptomatic control and has important clinical implications, particularly in elderly patients in whom prolonged non-steroidal anti-inflammatory drug use is associated with gastrointestinal, renal, and cardiovascular adverse effects [10]. These findings complement the improvements observed in VAS scores and patient satisfaction and further support the therapeutic augmentation of intra-articular HA therapy with triamcinolone acetonide and lignocaine. The observed between-group ROM differences (3.3-4.0 degrees across timepoints) are statistically robust but are of a magnitude comparable to typical measurement error in manual goniometry, particularly when performed by a single unblinded assessor without formal inter-rater reliability testing. This should be considered when interpreting these differences as evidence of clinically meaningful functional superiority rather than measurement variability.

The present study also found significantly higher patient satisfaction in the combination therapy group. Nearly 83% of patients reported excellent or good outcomes compared with approximately 61% in the HA-alone group. Similar associations between reduction in pain, improvement in function, and patient-reported satisfaction have been described in previous clinical studies evaluating viscosupplementation and intra-articular corticosteroids [11,12]. The higher satisfaction observed in our study is therefore likely a direct consequence of the earlier and more sustained symptomatic improvement achieved with combination therapy.

An interesting finding in the present study was the persistence of clinical benefit at 12 months despite a gradual increase in VAS scores after the three-month assessment. Altman et al. and Leighton et al. reported that although the effect of viscosupplementation gradually diminishes over time, many patients continue to derive clinically meaningful benefit for several months after treatment [13,14]. Our results are consistent with these observations, but the combination therapy group maintained superior outcomes throughout follow-up. This may indicate that effective suppression of early synovitis by triamcinolone allowed patients to derive greater long-term benefit from HA by interrupting the cycle of pain, inflammation, and functional limitation during the initial post-injection period.

Both treatment protocols were associated with a favourable safety profile with respect to the clinical adverse events assessed in this study; however, no structural outcome - radiographic imaging, MRI, or cartilage assessment - was collected at any time point, and the chondrotoxic potential of intra-articular lignocaine, together with published concerns regarding cartilage volume effects of intra-articular corticosteroid, was not evaluated. The safety conclusion should therefore be understood as limited to short-term clinical tolerability rather than confirmed structural safety. Post-injection knee pain, transient swelling, and local tenderness were the most common adverse events and resolved with conservative management. No cases of septic arthritis, allergic reaction, or other major complications were encountered. McAlindon et al. similarly reported that intra-articular HA and corticosteroid injections are generally safe when appropriate patient selection and aseptic techniques are employed [15]. Interestingly, post-injection knee pain was significantly less frequent in the combination therapy group in our study, probably because of the immediate analgesic effect of lignocaine together with rapid reduction of synovial inflammation following triamcinolone. Our findings are best interpreted against a guideline landscape that has itself been inconsistent on the role of HA. The American Academy of Orthopaedic Surgeons (AAOS) evidence-based guideline authored by Jevsevar did not endorse viscosupplementation for knee OA, citing insufficient supporting evidence at the time it was written [16]. The AAOS updated its guidance in 2021 (Third Edition), and this update did not move toward greater acceptance of HA: it reaffirmed - and downgraded in strength from strong to moderate - its recommendation against routine use of intra-articular HA for symptomatic knee OA, citing inconsistent results across high-quality trials [17]. The same update states that intra-articular corticosteroids may provide only short-term relief. Concoff et al. compared the minimum clinically important difference for pain relief across several nonsurgical interventions for knee OA and found that a number of injectable therapies met clinically meaningful thresholds, underscoring that the magnitude of treatment effect, and not statistical significance alone, should guide clinical decision-making [18]. Our finding of a treatment gap that widens rather than narrows over 12 months is therefore not straightforwardly explained by either the AAOS position or by the short-acting corticosteroid mechanism our own Introduction proposes, and the alternative explanations discussed above (operator effects, unblinded design, differential attrition) warrant serious consideration alongside a genuine treatment effect. Benchmarked against conventional minimal clinically important difference (MCID) thresholds for knee OA pain, as discussed by Concoff et al. [18], the 12-month between-group VAS difference of 2.0 points clearly exceeds these thresholds, while the six-week difference of 0.6 points falls below most published MCID values. The statement elsewhere in this manuscript that Group B showed significantly greater pain relief from the third week onwards should therefore be understood as statistically, but not uniformly clinically, meaningful across all time points.

The rationale for combining HA with an anti-inflammatory agent is further supported by recent reviews of the field. Glinkowski and Tomaszewski, in an umbrella review of systematic reviews and meta-analyses, concluded that intra-articular HA provides moderate but consistent benefit for pain and function, particularly in early-to-moderate disease, while also noting considerable variability across guideline recommendations that likely reflects differences in formulation, injection protocol, and patient selection among the primary studies reviewed [19]. Bruyère et al., restricting their umbrella review to placebo-controlled randomized trials, similarly found that the majority of high-quality systematic reviews confirmed a significant benefit of HA on both pain and function, and attributed earlier discrepancies in the literature largely to methodological heterogeneity rather than a genuine absence of effect [20]. This pattern is consistent with our own cohort, in which both groups improved, but the magnitude and durability of that improvement differed according to the specific injection protocol used, reinforcing the view that formulation and protocol details, rather than HA itself, may explain much of the inconsistency reported in the wider literature.

The broader concept of enhancing HA with an adjunct agent, rather than using it in isolation, is also gaining momentum. Fuggle et al., in an expert consensus statement, noted that intra-articular HA is increasingly being combined with other bioactive agents, including mannitol, chondroitin sulphate, tranexamic acid, and polynucleotides, with early evidence suggesting that such combinations may enhance efficacy beyond that of either constituent used alone [21]. Cao et al. reviewed both monotherapy and combination strategies for HA-based treatment of knee OA and reached a similar conclusion, identifying combination approaches, most often with platelet-rich plasma, as an area of active and promising investigation [22]. Our study differs from both of these reviews in the specific adjunct used: rather than combining HA with a newer biologic agent such as platelet-rich plasma or polynucleotides, we combined it with a corticosteroid and a local anaesthetic, both inexpensive and widely available agents. Direct comparison with biologic-adjunct combinations was not performed in this study, and no conclusions regarding relative efficacy, synergy, or cost-effectiveness between these approaches can be drawn from our data. A simple, low-cost combination of this kind may warrant further investigation, particularly in resource-limited settings where newer biologic adjuncts are less readily accessible.

It is also important to situate the present findings within the broader landscape of nonsurgical, image-guided interventions for knee OA, which includes platelet-rich plasma, prolotherapy, microfragmented adipose tissue, extracorporeal shockwave therapy, and other emerging modalities. These approaches vary considerably in cost, invasiveness, expected duration of benefit, and strength of supporting evidence. The present study did not include a direct comparison with any of these alternatives, and no claim of therapeutic equivalence with them can be made from our data. The relative role of HA combined with a corticosteroid and local anaesthetic, as evaluated here, within this broader treatment landscape requires dedicated comparative studies.

Compared with many previously published studies, the present investigation has several strengths, including a prospective design, a relatively large sample size of 320 patients, consistent injection protocols administered by experienced surgeons, clinician-measured and patient-reported outcome measures, and a 12-month follow-up. More importantly, while most published studies compared HA with corticosteroids as competing treatment options, the present study evaluated their combined use. This reflects routine clinical practice more closely and provides evidence that therapeutic augmentation of intra-articular HA therapy may offer earlier pain relief, improved knee ROM, reduced analgesic requirement, and sustained clinical improvement following a single intra-articular injection.

Limitations

This study has several limitations. First, allocation followed a systematic alternation method rather than formal randomization, and allocation was not concealed from the study team at the point of assessment; this introduces a potential risk of selection bias, and causal inference from these findings should be made cautiously. Second, between-group comparisons were performed using independent t-tests at each follow-up time point rather than a mixed-effects or repeated-measures model accounting for within-subject correlation across visits, and effect estimates with 95% confidence intervals were not computed, as the original patient-level dataset was not immediately available for reanalysis; approximately 30 unadjusted between-group comparisons were performed, which inflates the risk of at least one false-positive result and should be considered in mind for findings closer to p=0.05. We further note a minor discrepancy between the reported baseline VAS p-value and the value recalculable from the summary statistics presented; as the original dataset is no longer available for independent verification, the originally analyzed value has been retained. Third, the study was not prospectively registered in a clinical trials registry, as institutional policy at the time did not mandate registration for unsponsored, non-randomized studies; we recommend prospective registration for future studies of this nature. Finally, the study was conducted at a single tertiary care centre without radiographic or MRI assessment of structural disease progression. True randomized controlled trials with concealed allocation, appropriate longitudinal statistical modelling, multicentre design, and prospective registration are needed to confirm these findings. This analysis was conducted on a per-protocol basis, including only patients who completed the full 12-month follow-up (n=320); an intention-to-treat analysis incorporating the 34 patients lost to follow-up or excluded during the study was not possible. This may introduce attrition bias; in particular, protocol non-compliance was markedly higher in Group B than Group A (10/177 vs. 1/177; χ^2^=7.60, p=0.006, Fisher's exact p=0.011). A worst-case sensitivity analysis re-including these non-compliant patients as treatment failures showed the 12-month analgesic-use advantage in Group B remained significant (49.1% vs. 33.3%; χ^2^=8.47, p=0.004), whereas the difference in 'poor' satisfaction outcomes did not (11.7% vs. 9.5%; χ^2^=0.40, p=0.53), indicating the satisfaction finding is more sensitive to differential attrition than the analgesic-use finding. Because Group B received triamcinolone, lignocaine, and a greater total injection volume in addition to HA, and each surgeon consistently treated only one group, this study cannot isolate the individual contribution of each drug component from operator-related effects, and findings should be interpreted as reflecting the combined protocol as a whole. Additionally, Group B received a larger total injectate volume than Group A (6 mL vs. 4 mL); intra-articular volume and joint distension may independently affect pain or comfort, and this study cannot separate a volume effect from a drug effect.

Additional limitations include the absence of blinding of patients and outcome assessors, the lack of a placebo, sham-injection, or component-control arm (precluding conclusions regarding synergy or the contribution of any individual constituent), the non-usage of validated functional outcome instrument (e.g., Western Ontario and McMaster Universities Osteoarthritis Index (WOMAC), Knee injury and Osteoarthritis Outcome Score (KOOS), or Oxford Knee Score), non-standardized analgesic type and dosing, the potential for expectation and performance bias given the unblinded design, and a sample size not powered to detect rare serious complications; the theoretical chondrotoxic potential of intra-articular lignocaine and the physical compatibility of the injected mixture were not specifically assessed and warrant consideration in future protocol design. Analgesic use was recorded only as a binary daily requirement (yes/no) without capturing drug class, dose, or frequency, limiting the clinical granularity of this outcome. Baseline characterization did not systematically capture several potentially relevant confounders, including diabetes, cardiovascular disease, smoking status, prior knee trauma, meniscal or ligament injury, contralateral knee procedures, lower-limb alignment, activity level, or concurrent rehabilitation; this should be considered when interpreting the study population as having primary rather than secondary OA. Kellgren-Lawrence grading and goniometric ROM measurement were performed by a single assessor without formal inter-rater reliability testing, and rescue analgesic selection followed each treating surgeon's standard practice rather than a centrally standardized protocol. Injection speed and post-injection activity instructions (rest, weight-bearing, return to activity) were guided by routine clinical judgment rather than a standardized written protocol, which may have introduced additional unmeasured variability between patients and surgeons. Injections were performed using a landmark-guided anterolateral approach without imaging confirmation. Landmark-guided knee injections have a reported overall accuracy of approximately 82%, and anterolateral or anteromedial approaches specifically have been reported to be less accurate (67-75%) than a lateral mid-patellar approach (93%). Without imaging confirmation, some proportion of injections in either group may not have been truly intra-articular, which would dilute or obscure true treatment effects.

## Conclusions

This prospective comparative study showed that a single intra-articular injection of HA combined with triamcinolone acetonide and lignocaine was associated with significantly greater and more durable pain relief, functional improvement, and patient satisfaction than HA alone in patients with symptomatic primary knee OA, with benefits sustained through 12 months despite gradual attenuation in both groups after the three-month peak. Both protocols appeared safe within this cohort, with only minor, self-limiting adverse events and no major complications observed.

These findings suggest that an inexpensive corticosteroid-anaesthetic adjunct to HA warrants further investigation as a potentially low-cost alternative to combination strategies involving newer biologic agents, particularly in resource-limited settings, notwithstanding current guideline positions that continue to recommend against routine HA use; direct comparative and cost-effectiveness data are needed to substantiate this. HA combined with triamcinolone acetonide and lignocaine, using inexpensive constituent agents, may therefore be considered an effective conservative option for appropriately selected patients with symptomatic knee OA, although no formal cost-effectiveness analysis was performed.
